# Plague as a Soil Infection

**Published:** 1902-08-23

**Authors:** 


### PLAGUE AS A SOIL INFECTION.

ADDRESSING the British Medical Association Dr. Reginald Farrar summarised the evidence that outbursts of bubonic plague are dependent on the infection with a specific microbe of soil contaminated with the excreta of rats or other animals that have suffered with the disease, and that, except in pneumonic cases, infection is ordinarily the result of direct infection from the soil, the element of personal communication being a factor of relatively small importance. When we hear of the people of India perishing of plague at the rate of 10,000 or even 20,000 a week for months together, as has been the case more or less since 1896, it is difficult, he says, to believe—what is nevertheless the fact—that plague epidemics tend to remain strictly local. When we consider the appalling *intensity* of plague in India we cannot but be struck with the fact that its *spread* is in proportion significantly slow. A striking fact in dealing with plague epidemics is the success which attends the evacuation of the towns, and it is inconceivable that an epidemic dependent on personal infection, or indeed on any other than a soil or house infection, should cease, as has happened again and again, on the complete evacuation of the infected town, the people all the time being even more closely crowded than ever in the temporary camps. Striking as is the evidence as to the efficacy of evacuation in checking epidemics, yet more conclusive as to soil or house infection is the fact that such sporadic cases of plague as arise in health camps occur almost exclusively among persons who have surreptitiously visited their houses against orders. As to the manner in which the infection is conveyed to man Dr. Farrar is no less emphatic. The terrible susceptibility of the people of India to plague lies in the fact that they are, generally speaking, a barefooted race. The majority of them, moreover, wear toe-rings, which usually fit very badly and give rise to chronic abrasions. The women, again, use earth for scouring by hand their brass and copper vessels, and also sweep the floors of their houses with the bare hand, and it is a significant fact that women suffer from axillary buboes in a far higher proportion than men. Earth is also almost universally employed by the natives for a purpose for which sanitary paper is preferred in this country. To the well-shod and well-clad European, especially when putties are worn, plague is very little dangerous. It must be understood, however, that it is by rats and other rodents that localities become infected. On this all seem agreed. The question is how it is conveyed to man. For some time the theory that the infection is carried by fleas has been widely accepted. Lately, however, it has been shown that the fleas of man and of rats are of different species, and doubts having been expressed about the rat flea biting man the whole theory has been discredited. There is much then to be said for the soil infection theory as so ably set forth by Dr. Farrar.

